# "That never would have occurred to me": a qualitative study of medical students' views of a cultural competence curriculum

**DOI:** 10.1186/1472-6920-6-31

**Published:** 2006-05-26

**Authors:** Johanna Shapiro, Desiree Lie, David Gutierrez, Gabriella Zhuang

**Affiliations:** 1 Department of Family Medicine, University of California Irvine, School of Medicine, Rte 81, Bldg 200, 101 City Drive South, Orange, CA 92868, USA; 2 University of California, Irvine, Irvine, California, USA

## Abstract

**Background:**

The evidence is mixed regarding the efficacy of cultural competence curricula in developing learners' knowledge, attitudes and skills. More research is needed to better understand both the strengths and shortcomings of existing curricula from the perspective of learners in order to improve training.

**Methods:**

We conducted three focus groups with medical students in their first year of clinical training to assess their perceptions of the cultural competence curriculum at a public university school of medicine.

**Results:**

Students evaluated the informal curriculum as a more important source of learning about cultural competence than the formal curriculum. In terms of bias in both self and others, the cultural competence curriculum increased awareness, but was less effective in teaching specific interventional skills. Students also noted that the cultural competence curriculum did not always sufficiently help them find a balance between group-specific knowledge and respect for individual differences. Despite some concerns as to whether political correctness characterized the cultural competence curriculum, it was also seen as a way to rehumanize the medical education experience.

**Conclusion:**

Future research needs to pay attention to issues such as perceived relevance, stereotyping, and political correctness in developing cross-cultural training programs.

## Background

Cultural competency instruction in medical education has been the thrust of several recent reports in the US and the UK [1-3]. Evidence is growing that improving cross-cultural communication skills in healthcare providers is associated with better patient outcomes [4,5] and has the potential to reduce health disparities [6] and improve access to care [4]. However, a recent systematic review evaluating cultural competence training of health professionals concluded that lack of methodological rigor made it difficult to draw conclusions regarding the effectiveness of specific educational interventions [7].

Attitudes of students toward diversity training are equivocal, although survey-type methodologies tend to report positive attitudes toward diversity training [8,9]. In another study, residents lamented their lack of "formal" education on this topic, and felt they were poorly prepared to deal with cross-cultural clinical encounters [10]. Similarly, a different investigation concluded that much of learners' self-reported cross-cultural competency came from their own improvisational coping [11]. Yet learners were also concerned that formal cross-cultural education might result in stereotyping of different racial and ethnic groups; and in any case was excessively abstract and theoretical [12].

While some research has provided evidence that medical students' knowledge, attitudes, and skills can be positively changed as a result of participation in a cultural competency curriculum [13,14], other studies suggest that exposure to a cross-cultural curriculum has little or no effect on student skill acquisition [15,16]. Further, even when learners report having adequate cross-cultural communication skills and training, they feel that both systemic and patient factors inhibit their providing quality care [12].

We still do not have sufficient information from the perspective of students regarding their cultural competence curricula. The purpose of this study was to qualitatively assess the views of third year medical students at a public university school of medicine regarding its cultural competence curriculum (CCC), which incorporated an integrated longitudinal approach [17], the concept of cultural humility [18,19], and specific cross-cultural communication tools [20].

## Methods

Participants were 16 third year medical students, 17.4% of the class. There were 6 males and 10 females (62.5%), a somewhat higher representation of women than in the student body as a whole (50.0%); and 17.7% underrepresented minorities, compared to 10.0% overall. Students were recruited via a series of emails as well as personal invitation. Students were offered the incentive of a free lunch, and participated in a raffle for which the prize was a medical textbook. Sampling was in part purposive, in that the first two authors made an effort to attract students with a wide range of attitudes toward cultural competence training, including students with intense feelings about the subject, both positive and negative, and students with less interest in the topic. Sampling was also a matter of convenience. Because of the relatively small response, we accepted all students who volunteered.

Nineteen students responded affirmatively. Two could not attend the focus groups due to scheduling conflicts, but agreed to participate in individual interviews. Three others could not arrange either option. The three focus groups contained respectively 5, 5, and 4 students. All participating students had had a high degree of contact with patients from different cultural backgrounds. This study was approved by the university's human subjects institutional review board. Following the requirements of that approval, the nature and purpose of the study was carefully explained in the recruitment email. The study was again explained at the start of each focus group, including issues of anonymity and confidentiality regarding tape-recording and transcription of tapes, as well as reporting of findings; and verbal consent was obtained from group participants. Since consent was inferred from response to the recruitment email (students could refuse to participate simply by not replying) and to the oral presentation of the study by the faculty member, our IRB considered verbal reiteration of consent sufficient.

A preliminary version of the schedule of focus group questions was developed by a researcher (JS) who had previously conducted focus group research on cultural competence with other medical learners [12,21]. This interview guide was reviewed and modified by the second author (DL) who directs the Patient Doctor course in which some of the Cultural Competence Curriculum was embedded. The groups followed standard focus group methods, including creating an informal atmosphere, allowing conversation to flow naturally rather than adhering to a strict schedule, encouraging differences of opinion, and eliciting comments from silent members [22]. The location of the focus groups was a small, comfortable conference room. JS was the primary moderator and an assistant moderator (an undergraduate student [DG]) made process notes and observations, as well as asked clarifying and supplemental questions as needed. Each group was approximately 75 minutes in length and was tape-recorded. DG conducted the individual interviews by telephone. These interviews used the same interview guide prepared for the focus groups, and lasted about 20 minutes. They were not tape-recorded, but the interviewer typed extensive notes during the interview, as well as made summary notes immediately afterwards. All recordings were transcribed verbatim and these texts, in addition to the group process notes, and written notes from the interviews, provided the basis for data analysis.

### Data analysis

As is typical in qualitative research, data gathering and data analysis occurred to some extent simultaneously, in the sense that the two moderators discussed their impressions of each group immediately upon its completion and adapted the interview guide to better reflect student concerns [23]. JS kept notes of these debriefings to establish an audit trail. We achieved data triangulation by referring to the audiotapes, transcriptions, debriefing notes, and analyses of the transcripts prepared independently by each member of the research team [24]. We incorporated investigator triangulation in the analysis phase due to the range of perspectives and experience represented among the researchers (physician, psychologist, pre-medical student, and post-graduate sociology major). We included member checking in that at the end of each focus group, JS summarized the main points of the discussion, and asked group participants to confirm and/or modify this summary [25].

The independent transcript analyses prepared by each researcher first coded key words and phrases based on the interview guide, then reexamined these to generate new categories that eventually produced essential themes [26]. JS integrated these analyses into a comprehensive synopsis which was shared and discussed by all researchers. We considered criteria of frequency, extensivity, and intensity in evaluating the data. Our goal was to identify recurring patterns and comparisons across groups, while simultaneously acknowledging outliers and disconfirming evidence. Resolution of disagreements was obtained through face-to-face and email discussion. The themes and interpretations reported below represent the results of our consensual process. [27].

## Results

### Formal vs. informal curriculum

All of the focus groups spontaneously noted that the CCC consisted of both formal and informal elements. Formal elements included a required reading assignment [28], standardized patient modules containing a cultural component, and lectures. The informal curriculum included learning about cultural competence from residents, attending physicians, and patients in clinical situations as well as from the diversity of fellow students.

Overall, most students evaluated both the formal and the informal CCC as useful and relevant. Of note is that the two students interviewed separately were more negative about the CCC. One student described the formal curriculum as "not that great, superficial, it didn't provide any concrete skills. There was nothing new. Honestly, I was very frustrated with the superficiality."

Most students considered the informal curriculum to be superior to the formal curriculum in terms of teaching cultural competence because of the direct link it provided between knowledge presentation and skill acquisition. As one student expressed it, "I think the greatest part [of CCC] is seeing by example how to act, or act in a certain situation." Another student said, "I'm learning more on the go [about CCC], in clinic from my preceptor. And I've learned more in the clinic actually than I ever did in any lecture that I had, if I think back." A third student commented: "I guess what I'm trying to say is that you really learn when you're thrown in these situations. Like right now...I'm learning for the first time about Vietnamese and their culture and just different ways that they need to be treated as a patient, things you need to be sensitive about. But it's only because I'm working with these patients."

However, several students noted that attending physicians rarely had time to talk about cultural issues and even more felt that opportunities for clinical teaching about cultural issues in the patient environment were significantly underutilized. Students reported that while some of their best, most practical cultural competence teaching took place in the clinics or hospital, these experiences happened randomly, varying significantly from one rotation, one clinic, or even one resident or attending to another. As one student noted, "I'm sure I do [make culturally insensitive mistakes], but I don't know what they are because no one's ever watched me and said you're doing this and how to be constructive about it."

Many students mentioned that one of the strongest aspects of the informal curriculum was the diversity of the student body. They seemed to feel that, because they were surrounded by diversity, they could not be biased. As one student put it, "A different generation might not have had this advantage. But young people nowadays, we're all so diverse, we're used to being with people from other backgrounds since elementary school, so it's no big deal."

### Awareness vs. intervention regarding bias

As the above quotation suggests, it was initially difficult for students to acknowledge self-awareness as relevant to culturally competent patient care. Students described self-awareness exercises as "a waste of time" and "too touchy-feely." After some probing, however, several students were able to give specific examples of how they had become aware of their own biases as a result of exposure to the CCC. One student (himself Vietnamese) commented that the CCC had enabled him to develop a more professional perspective: "And that [the CCC], when I was talking with them [Vietnamese patients], kept me from like... just laughing you know, making an inappropriate response when they would express these unscientific beliefs." In the words of another student: "... [This was] something that the [CCC] originally made me aware of, because that never would have occurred to me [i.e., the possibility that she could have personal prejudices]. I think that I'm an empathetic person and that I would go into every encounter with an open heart but as it turns out I really do have a harder time with certain patients."

Students also commented that faculty and residents sometimes demonstrated stereotypic, denigrating attitudes toward patients of a specific ethnicity. "Some residents talk about patients doing the Macarena, which means Latina women in labor who are loud." Examples of unfair *positive *bias in faculty were also observed: "Sometimes you see, like examples of physicians who are kind of more biased toward patients who are of their own culture. I remember this one rotation where this doctor would spend half an hour on one patient that was of his culture, the whole time speaking that language... and then in comparison he would spend 5 minutes with patients of various different cultures..."

Students had no difficulty identifying such examples as race- or culture-based stereotyping, and attributed part of this awareness to learning that occurred in the CCC. But they varied in their ability to *address *or respond to these situations. The majority felt similarly to a student who said she found it impossible to say anything to faculty or residents who made culturally insensitive remarks because the situation was too "intimidating." These students were concerned about the possible adverse effects of action on their course evaluations and/or grades. On the whole, students across the three groups did not feel well-prepared to deal with such culturally biased statements in clinical contexts.

### The search for cultural "balance."

Many students wanted group-specific knowledge about as many different cultures as possible. In the words of one student, "I always feel like part of my knowledge is lacking... I know I should be sensitive to different things of their healthcare but I don't really know what they are..." A student from a different group said, "I mean I know the task may be very daunting, but it would be insightful if I knew about as many cultures as possible..."

However, students also expressed concern that such presentations might stereotype different ethnicities. One student said, "Sometimes I think it's better when I go into a [n exam] room and figure the patient out for myself without any preconceived notions of 'Oh, this patient is Hispanic so she's going to act this way."' Another noted, "As soon as you start you know, painting a certain ethnicity as, you know, look out for these, you're basically saying every person who is this ethnicity has this."

These students expected the CCC to help them achieve a "balance" between acquiring appropriate cultural knowledge and approaching each patient as an individual, but they were often disappointed. One student summarized this point of view in the following way: "To me it's hard to find the balance, because we're told on one hand that you treat all these patients as patients, you know it doesn't matter where they're from... On the other hand, we're told if they have a certain ethnicity there are all these other things we need to think about."

### Did the CCC promote political correctness?

There was a difference of opinion about the openness of the student body to discussing sensitive issues of culture. On the one hand, some students' perception was that their peers felt comfortable expressing their cultural and religious beliefs. "My experience is that people have been themselves and let their religion or their culture or whatever, come out." However, other students felt that the whole discussion of culture was overlaid by a strong degree of political correctness, so that it was difficult for people to state their true opinions or feelings. "We're all taught to be so politically correct these days and no one wants to say how they really feel. Like it was hard for me to admit I had certain biases about certain cultures but I do..."

### The CCC and humanism

Several students were concerned about the decline in humanistic values during their third year. In the words of one student, "When I go home I just don't think about patients, I think about their medical issues. There have only been a few times when I actually start to think about what I call 'personal connection,' where I go you know I wonder how it's affecting their life, I wonder how they're really feeling."

As a kind of antidote, students felt it was important that "... [The CCC] showed that the medical school actually cares about culture and people, you know." The implication was that the CCC could reinvigorate students with a more patient-centered approach.

Finally, although these students all valued the concept of cultural competence, none suggested that we test for this competency through either written or clinical examination.

## Discussion

Several aspects of these discussions with third year medical students were illuminating. One was the strong preference expressed across all three groups for the majority of the CCC to be integrated into the informal curriculum, especially during the third year of training, when students start their clinical rotations. Students appeared to feel that they learned best in this type of integrated environment, where knowledge had immediate and direct application. Although this is an attractive proposition, it faces the perpetual challenge of how to systematically train large numbers of attendings and residents in all the steps of cultural awareness, sensitivity, knowledge, attitudes, and skills; and how to simultaneously attend to medical and cultural issues in any given patient encounter.

Also of concern to medical educators may be students' tendency to minimize the importance of self-awareness in developing cultural competence. This finding was reported almost a decade ago [17] and confirms the difficulty of convincing students that they need to carefully reflect on and confront their own potential for bias. Students seemed to find reassurance in the fact that they were training with culturally diverse peers. However, there tends to be more homogeneity than heterogeneity in terms of academic ability and socioeconomic status in medical school. In addition, the medical school experience is known for its capacity to "stamp out" the diversity of those who succeed in matriculating [29].

Although the CCC was helpful in making students aware of expressions of bias in others, it was less effective in giving them useful tools for addressing these situations. This finding suggests the importance of providing students with concrete, skill-based training in confronting bias, while ensuring that the medical education system provides protections for students who exercise such skills.

Students did not appear to have theoretical models other than cultural difference within which to understand the relationship between doctors and patients. This dearth of explanatory mechanisms may have contributed to students' confusion about how to "balance" knowledge of specific cultural groups with respect for individual differences. One partial solution to this dilemma might be greater attention to the concept of cultural humility [30]. Although our curriculum already included such exposure, it appeared students by and large ignored its implications. This suggests the need for more explicit and systematic training to enable students to adopt a position of respectful curiosity about a person's background, regardless of his or her particular culture, thus positioning themselves as learners and their patients as teachers. This approach also has the advantage of helping students be aware of and partially rebalance power dynamics within the doctor-patient relationship [18].

Because of differences in opinion as to whether the CCC operated in a context of political correctness, it was unclear to what extent students either confronted their own biases or developed respectful understanding of culturally different perspectives. While some participants maintained that students could "be themselves" and express opinions openly, a significant minority maintained that they were constrained by certain norms that made it unacceptable to verbalize culture-based negative attitudes. Such beliefs could seriously impede the ability of the CCC to penetrate more than superficial levels of student attitudes, leading to external conformity to perceived norms rather than genuine respect and understanding of difference. Occasionally, an article raises questions regarding whether political correctness can compromise the quality of physician training or patient care [31], and it seems a more open discussion of this issue is needed.

Despite concerns about the CCC, many students regarded it as a method for humanizing their medical education. This link between the CCC and humanism is an interesting one, and has been noted by others. For example, one author suggests that culturally competent care and patient-centered care (based on several humanistic properties) are philosophically similar and both contribute to patient empowerment [32] It is possible that the CCC helped connect students to the life stories of their patients, particularly as these were manifested in the informal curriculum, and this experience had a rehumanizing effect.

The fact that no student advocated assessment of the cultural competence curriculum deserves some reflection as well. This omission may mean nothing more than students' understandable aversion to yet more examination in an already test-intensive academic environment. It may also intimate that students do not perceive cultural competence as "real learning" on a par with the basic sciences and clinical knowledge. Students may also see cultural competence in much the same way good communication used to be viewed, as something one either has or doesn't have. Finally, it is possible that students' attitudes toward assessment reflect those of many medical educators who also have concerns about how to meaningfully conduct evaluation of culturally competent knowledge, attitudes, and skills [33].

### Limitations

We were disappointed that despite determined recruitment, only a small percentage of the class became involved in this project. We believe that while we were successful in attracting both students who cared passionately about the CCC and those who held significant reservations, we had fewer participants representing the "silent majority." Further, the students who participated appeared to hold fairly positive views about their cultural competence training, in contrast to the significantly more negative perceptions shared in the two individual interviews. The focus groups might have attracted more pro-social, "helpful" types of students who have inherently positive views of the educational system. The two students in the individual interviews, by contrast, might have been outliers who chose to express views privately that they felt to be at variance with group norms. Nevertheless, the diversity of opinions expressed during the group discussions provided some reassurance that, despite the small number of students, we did succeed in sampling widely differing viewpoints about the CCC.

## Conclusion

Students in this study wanted more integration of cultural competence training in the clinical setting as part of the informal curriculum. They also needed more specific assistance in learning how to deal with situations that expressed prejudice or bias. Further, students hoped for better guidance in achieving a balance between learning about specific cultures while respecting individual differences. Despite some concerns as to whether political correctness permeated the CCC, it was also perceived as one way of rehumanizing the medical education experience.

## Abbreviations

CCC – Cultural Competence Curriculum

IRB – institutional review board

## Competing interests

The author(s) declare that they have no competing interests.

## Authors' contributions

DL and JS developed the initial conceptualization of the study and worked together to develop the research questions and the question route. DL developed the participant recruitment strategy, which was implemented by DG. DG conducted the literature review under the guidance of DL and JS. JS conducted all focus groups, and was assisted by DG, who also took observational field notes during each session. DG also conducted the two individual interviews. JS and DG debriefed together after each focus group. DG transcribed all group and individual interviews. JS formulated the data analysis plan, and all authors reviewed and prepared theoretical notes on all transcripts. Each author prepared an initial analytic summary, which were then integrated by JS. All authors reviewed and modified a final interpretive summary. JS prepared the initial draft of the manuscript, which was revised by DL, DG, and GG. All authors read and approved the final manuscript.

## Pre-publication history

The pre-publication history for this paper can be accessed here:


